# Supplementary material for: Engaging citizens in local health policymaking. A realist explorative case-study
Source: PLoS One. 2022 Mar 24;17(3):e0265404. doi: 10.1371/journal.pone.0265404 (PMC8946671; doi:10.1371/journal.pone.0265404)
Supplement: S1 Appendix — (DOCX) [file pone.0265404.s001.docx]

**Appendix S3: Appendix I: Summary of CMOs**

| **Residents’ health policy priorities stem from their holistic experiences and perceptions and differ from professionals’ priorities** | | | | | | |
| --- | --- | --- | --- | --- | --- | --- |
|  | **Context** | | **Mechanism** | | **Outcome** | |
| 1. | Residents experience a lack of suitable and affordable housing especially for elderly residents | | Elderly residents wish to remain within their own villages and close to their own social network | | Residents want more housing which enables elderly residents to live independently in their own homes and communities for as long as possible | |
| 2. | - Residents enjoyed living in their village due to their roots and social network. - None of the newly built housing was designated as social housing | | They worried that the new housing was unaffordable for local residents and felt this was unfair to local residents | | They worried that due to the unaffordability of housing and the increasing housing shortage, an increasing number of local residents would be forced out | |
| 3. | - Rural area with fragmented health and care services, especially regarding elderly care & carers’ support. - Neither primary care services nor municipality were easily accessible for carer and none had clearly communicated available services | | Because carers felt left to their own devices and that they had to figure all the options out themselves, they experienced the system as stressful | | Finally, they had found suitable care for their elderly parents, but they had experienced it as very stressful as it took a lot out of them | |
| 4. | - Few opportunities to exercise outside or to meet up socially (e.g. few public pedestrian or bike paths). Furthermore, on many of the existing paths, residents experience inconvenience & harassment due to heavy drinkers. - The municipality developed a plan to implement beaches public, however, the local residents who live there do not want to offer up their plots of lands | | Residents feel the need for outside social meeting spaces as they think this would improve social cohesion and want the municipality to maintain law & order on current paths | | Municipality’s plan for public beaches is seen as improbable and would prefer the municipality to maintain current law & order and to implement new paths | |
| 5. | Remote village with limited and infrequent public transport increases dependence on owning a car | | As such residents worry that this increases their loneliness and isolation | | This makes more remote villages less popular | |
| 6. | Suitable and accessible primary care services within village | | Access to suitable care services makes it easier to live within the villages as residents do not need to travel to the nearby city. | | This helps to ensure residents want to stay within the village | |
| 7. | Small rural village with limited social activities and meeting spaces for children aged 12-18 years old | | Children find living in the village boring as there is little for them to do without such meeting spaces and activities | | Now they organize parties (often with alcohol) within their own sheds to combat boredom | |
| **Residents felt ownership for enabling residents’ engagement within the community by organising social activities and feeding back residents’ needs and experiences to the municipality** | | | | | | |
|  | | **Context** | | **Mechanism** | | **Outcome** |
| 1. | | Residents involved in the church parish | | Feel a desire to be involved because of their sense of community, social responsibility and reciprocity | | Being involved in such a way provides them with a social network and fun |
| 2. | | Residents had started project implementing Automatic External Defibrillators (AED) in the village & the wider area and some did so because of their families’ history of heart problems | | Some felt motivated to start the project partly because of family members, but most because they wanted to bring neighbours together | | - The project was successful which created a lot of goodwill amongst neighbours - It also provided neighbours with good links with municipality professionals |
| 3. | | When the Board was first set up, the municipality and the Advisory Board had to find suitable & successful ways to collaborate | | Slowly the Board’s role has shifted to engage other (not-yet engaged) residents. Furthermore, the Board feels like they are a linking-pin between the communities and the municipality. | | While the Board and the municipality are still exploring their roles & opportunities for collaboration, the Board feels as if the municipality has started listening more to the Board |
| 4. | | Both community-led initiatives as well as organisations (e.g. the municipality, community services) are increasingly seeking volunteer input and support on a wider range of themes (e.g. buddies for lonely residents). However, there is a smaller pool of available volunteers | | Community services often asks for volunteers, but with community services (which are more professionally structured and funded by the municipality) residents do not feel triggered/motivated to help out as there is no sense of community or reciprocity | | Organisations, like community services, might have more success in recruiting volunteers if they provided some form of compensation (e.g. small stipend) |
| **Residents need the municipality to better facilitate their involvement by improving their communication and accessibility for residents and community-led initiatives** | | | | | | |
|  | | **Context** | | **Mechanism** | | **Outcome** |
| 1. | | Significant reduction in the number of volunteers involved in community-led initiatives | | Residents have increasingly less time and feel less motivated to volunteer besides their jobs and other day-to-day tasks | | - This makes it harder to keep community-led initiatives going. - Volunteers wanted the municipality to provide them more support in recruiting new volunteers |
| 2. | | Residents often have ideas for practical projects (e.g. building their own skateparks, or playgrounds) but have previous negative experiences of collaborating with municipality | | Previous negative experience makes residents feel like the municipality would not listen to them and their ideas anyway. They worried that municipality would get bogged down in bureaucracy | | - This keeps residents from acting on their practical project ideas - Residents do not ask the municipality for support and do not start new projects |
| 3. | | Municipality does not communicate often enough with community-led initiatives | | When municipality reaches out to engaged residents and meets them on the times & locations when residents meet, then the municipality shows willingness to listen and offers residents the chance to collaborate more often | | This would lead to shorter communication lines and provide residents the chance to ask the municipality questions on the spot |
| 4. | | Volunteers are required to follow a lot of municipal and bureaucratic rules. Especially when looking for and applying to funding opportunities (e.g. for improving sustainability, social activities) | | Volunteers experience such bureaucratic tasks as demotivating as it is not their main motivation for being engaged | | This is why they want the municipality to actively reach out to community-led initiatives and better communicate such opportunities. |
| 5. | | The municipality brings out a monthly free newspaper, directly delivered to residents’ houses. However, this free newspaper does not contain any information regarding available services, funding or volunteering opportunities. | | Residents are not motivated enough to seek such information (regarding available services or funding, volunteering opportunities) out themselves. | | Participants felt the free newspaper should be updated to include such information |
| 6. | | Municipality has an information centre for community-led initiatives | | Residents feel frustrated because the information centre does no outreach and does not provide information that residents feel they need | | Residents would prefer the information centre take a more active role in, e.g. helping them recruit more volunteers |
| 7. | | Municipality is increasingly involving village councils and church councils in the development of municipal policies and plans | | Because the municipality is increasingly reaching out to the councils, residents are feeling more heard and valued by the municipality | | Residents are increasingly seeing the benefit of collaborating with the municipality |
